# Pleasant body odours, but not genetic similarity, influence trustworthiness in a modified trust game

**DOI:** 10.1038/s41598-020-60407-6

**Published:** 2020-02-25

**Authors:** Janek S. Lobmaier, Fabian Probst, Urs Fischbacher, Urs Wirthmüller, Daria Knoch

**Affiliations:** 1 Department of Social Neuroscience and Social Psychology, Institute of Psychology, University of Bern, Bern, Switzerland; 2 Department of Child and Adolescent Psychiatry, University of Zurich, Zurich, Switzerland; 3 Department of Economics, University of Konstanz, Konstanz, Germany; 4 Thurgau Institute of Economics, Kreuzlingen, Switzerland; 5 Universitätsinstitut für Klinische Chemie, University of Bern, Bern, Switzerland

**Keywords:** Decision, Human behaviour

## Abstract

Identifying trustworthy partners is an important adaptive challenge for establishing mutually cooperative relationships. Previous studies have demonstrated a marked relationship between a person’s attractiveness and his apparent trustworthiness (beauty premium). Kin selection theory, however, suggests that cues to kinship enhance trustworthiness. Here we directly tested predictions of the beauty premium and kin selection theory by using body odours as cues to trustworthiness. Body odours reportedly portray information about an individuals’ genotype at the human leucocyte antigen system (HLA) and thus olfactory cues in body odours serve as a promising means for kin recognition. Ninety men played trust games in which they divided uneven sums of monetary units between two male trustees represented by their body odour and rated each body odour for pleasantness. Half of the odours came from HLA-similar men (suggesting closer kin) and half from HLA dissimilar men (suggesting non-kin). We found that the amount of money the players transferred was not related to HLA-similarity, but to the pleasantness of the trustee’s body odour. By showing that people with more pleasant body odours are trusted more than people with unpleasant body odour we provide evidence for a “beauty-premium” that overrides any putative effect of kin.

## Introduction

Trust is essential for establishing and maintaining mutually cooperative relationships. Selecting trustworthy partners is hence very important for successful navigation in our complex social world. Kin selection theory^1^ proposes that we should trust our kin more than we trust strangers, whereas the “what-is-beautiful-is-good” stereotype^2^ predicts that attractive people are generally trusted more than people who are less attractive. Previous work on visual appearance has found evidence for both hypotheses. Specifically, some studies found that we trust people more if they look similar to us, while others reported that good-looking people are trusted more than less good-looking people. However, these assumptions have not yet been systematically tested against each other. In the present study, we compared the relative influence of kin selection and the beautiful-is-good stereotype on human trust decisions by using peoples’ body odours as indicator of their trustworthiness. Body odours reportedly play a role in kin recognition^3^ and they can be more or less pleasant. In addition, odours have been found to modulate interpersonal trust^4^. Body odours hence offer a promising means to test to what extent people base their decisions about whom to trust on genetic similarity or on odour pleasantness.

According to kin-selection theory^1^, levels of prosocial behaviours such as trust or generosity should be higher between individuals who are genetically related. Indeed, effects of genetic relatedness on (pro-)social behaviour have been documented in several species, ranging from social insects^5^, to birds^6^, rodents^7^ and primates^8^. Also in humans, prosocial behaviour is more likely to be observed among kin^9–12^, but it extends to genetically unrelated friends and even strangers. It is yet unclear to what extent the genetic similarity between two individuals has an effect on whether they trust each other or not.

One way of assessing one’s relatedness to other group members is through phenotype matching which operates by trait-based assessment of phenotypic similarity. Facial resemblance is a likely candidate on the basis of which humans may recognise kin^13^. Indeed, participants have been shown to trust others more in a trust game, if the trustee’s face showed visual resemblance to the truster’s face^14,15^. Another study found that contributions in a public goods game increased as a function of facial resemblance between the players^16^. A limitation of these studies is that kinship was manipulated indirectly by altering facial self-resemblance using computer graphics software, which is a rather artificial manipulation of genetic similarity^14^.

A promising way to directly study influences of genetic similarity on social decisions is by using body odours. Indeed, kin recognition has been shown to operate via olfactory cues in body odour^17–19^. Body odours have been found to portray information about an individuals’ genotype at the major histocompatibility complex (MHC, or human leucocyte antigen system, HLA in humans, for a review see^20^). The MHC is a large chromosomal region containing highly polymorphic genes which play a central role in the process of adaptive immunity^21,22^. A large body of research suggests that discrimination between genetically similar and dissimilar others is achieved via odour mediated MHC-similarity (for a review, see^23^). Particularly relevant for the present study are two neurophysiological studies showing that humans possess the sensory capacity to recognize the presence of HLA-associated olfactory cues^24,25^. Notably, recent work suggests that the human olfactory system is far better than commonly assumed^26,27^.

A second line of research has demonstrated a marked relationship between people’s attractiveness and how trustworthy they appear. For example, Wilson and Eckel^28^ showed that good-looking trustees were viewed as more trustworthy in a game involving trust and reciprocity. A similar “beauty premium” was found in a hypothetical trust game, where participants invested significantly more money in good-looking partners than they did in partners that were less good looking^29^. An evolutionary account of this “beauty premium” states that attractiveness benefits can be explained by mating motivations^30^. However, because the beauty premium also affects same-sex interactions^29,31^, others have pointed out that mate choice is not the only type of partner choice that has benefitted from preferences for physical attractiveness^32,33^. Other factors such as (intra-sexual) competition, friendship or leadership selection can likewise play a role in bias towards attractive individuals.

The present study is designed to test the relative influence of kin selection and beauty premium on trusting behaviour by using body odours as cues to someone’s apparent trustworthiness. Specifically, we examined to what extent peoples’ trusting behaviour is influenced by odour-mediated HLA-similarity or by how pleasant they find the body odour. We assessed the genetic similarity between the interaction partners by typing all participants at six HLA loci. Participants played a modified trust game in which the recipients were represented solely by their body odour. That is, the recipients’ body odours were collected from their armpits using cotton pads and were presented to the participants in glass jars. These body odours either stemmed from an HLA-dissimilar individual, or from an HLA-similar individual, hence the genetic similarity was real rather than manipulated as in the face resemblance studies reported above. To test whether trusting behaviour is related to body odour pleasantness, participants were subsequently asked to rate how pleasant they found the body odours.

**Figure 1:**
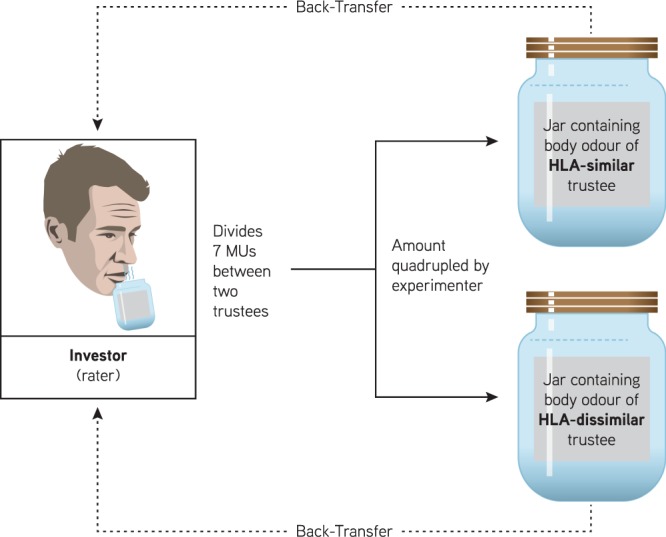
Round in the Trust Game: The investor (rater) is asked to divide 7 MUs between two trustees who are represented solely by their body odour. One trustee had a HLA that was similar to the investor, the second trustee was HLA dissimilar. The amount transferred to the trustees was quadrupled by the experimenter. Trustees could back-transfer half of the received amount to the investor, or he could keep everything to himself. Rounds in the Allocation Game were the same except that the transferred amounts were not quadrupled and there was no back-transfer.

Participants (all male) decided how many monetary units (MUs) they want to invest in two different trustees. They were asked to divide 7 monetary units (MUs) among two recipients (also male), one of which was HLA-similar, the other HLA dissimilar. This amount was then quadrupled by the experimenter and each trustee could decide to back-transfer half of the total amount or to keep all to himself (see Fig. 1). Critically, in this trust game, the participant faced real consequences: If he trusted the wrong person he would end up with no money, if he gave money to a trustworthy person he would end up with more than he transferred. Hence, in this trust game, investor’s decision whom to trust has direct consequences for the investor himself. Note that the trustees’ decisions were recorded beforehand, since they were represented only by their body odour during the trust game. Specifically, the trustees were asked whether or not they would return half of the transfer they received. To scrutinize whether real consequences are critical for such resource-distribution decisions, participants additionally played a simple allocation game in which they were again asked to divide 7 monetary units (MUs) among two recipients (one HLA-similar, one HLA dissimilar), but without the possibility of any back-transfer (i.e., without any consequences for the participant). Here he acts as a benefactor by distributing money that he will never see again. The pairings of the odours were different than in the trust game but were made up from the same four odour pads as in the trust game.

Above mentioned work puts forward two hypotheses that help explain strategies for deciding how to divide resources between two individuals: People might rely on cues to kinship or they may use a “beauty premium”. These hypotheses are not mutually exclusive but make distinct predictions. The present study was designed to specifically test whether social decisions (whom to trust and how to divide resources) are driven by cues to kinship or by pleasantness or by both. If kinship-recognition plays a role, we expect that HLA-similar individuals are favoured over HLA-dissimilar individuals. If social decisions are made based on the beauty-premium, we expect individuals with a body odour that was perceived as being pleasant to be favoured over individuals with a less pleasant body odour. Because in the trust game participants’ decisions have direct consequences for the investor, while the allocation game measures how people distribute a fixed amount of resources without having to fear exploitation, we might expect the divisions to differ in the two games, such that genetic similarity might play a larger role in the trust game than in the allocation game. To reduce the potential influence of mating motivations in the decisions whom to trust more (for a review, see^30^), we tested only men: All body odours were male and they were evaluated by men.

## Statistical Analyses

Statistical analyses were performed using SPSS 25.0 and the level of significance was set at *p* < 0.05. To test the hypothesis that HLA similarity influences how much money the odour donors were entrusted with, we first assessed whether more money is allocated to the HLA-similar than to the HLA-dissimilar trustee using paired t-tests.

In a second step, we used multilevel linear regressions to further analyse the relationships between HLA similarity and the amount of money that was transferred to the trustees, and to accommodate the influence of pleasantness and intensity of the the body odours. For this, we calculated difference scores between the amount allocated to the HLA-dissimilar trustee and the amount allocated to the HLA-similar trustee (i.e., “difference amount transferred”) in such a way that a positive difference score signifies that more money was given to the similar odour donor and a negative score means more was allocated to the dissimilar odour donor. We also calculated the difference between the number of alleles shared between HLA-similar odour donors and the raters and the number of alleles shared between HLA-dissimilar odour donors and the raters. Finally, we also calculated difference scores between the rated odour pleasantness and intensity of HLA-similar and dissimilar donors. We ran multilevel linear regressions with “difference amount transferred” (i.e., difference scores between the amount allocated to the HLA-dissimilar trustee and the amount allocated to the HLA-similar trustee) as dependent variables, separately for the two games. The first model included HLA-difference-scores as Level-1 predictors of MU allocation. Raters were entered at Level 2 as random factors. In a second model, we included pleasantness-difference-scores together with HLA-difference scores at Level 1 (fixed factors). In a third model, we controlled for odour intensity by entering intensity-difference-scores in addition to pleasantness- and HLA-difference scores (fixed factors).

To explore whether pleasantness ratings were related to HLA-similarity, we ran an additional multilevel linear regressions with pleasantness difference as dependent variable and similarity difference as predictor.

The reported estimates in the multilevel models are unstandardised regression coefficients.

## Results

Of the 96 men initially participating as odour raters, five later decided to withdraw from the study and one was sick on the day of the rating session. The final sample of odour raters hence consisted of 90 men. A total of 180 rounds were played in the trust game (2 ×90), 180 rounds were played in the allocation game (2×90) and a total of 360 (90 ×4) ratings were completed. In 16 ratings (4.5%), participants indicated that no odour was perceivable. We note that the non-perceivable trials were not always from the same pad (i.e., from the same man). In other words, there was no pad that was not perceivable in all cases: the non-perceivable pads did not come from specific men, but were randomly distributed over different donors. Results remained the same whether these trials were included or not, we hence report data including all trials.

### Trust game

Paired t-tests revealed that there was no significant difference between the amount of money transferred to the HLA-similar and HLA-dissimilar donor (*M*_*similar*_ = 3.656, *SD*_*similar*_ = 1.423, *M*_*dissimilar*_ = 3.344, *SD*_*dissimilar*_ = 1.423, *t* = 1.037, *df* = 89, *p* = 0.303).

**Figure 2:**
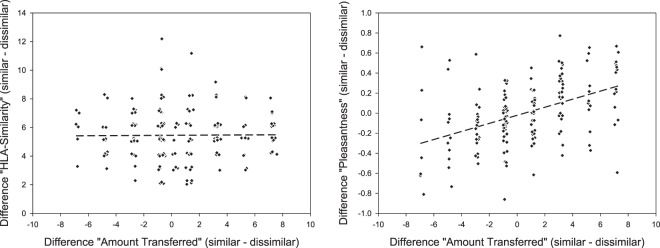
The relationship between HLA-similarity and the amount transferred to the trustee in the Trust Game (left panel), and between the rated pleasantness of the body odour and amount transferred to the trustee (right panel). Difference scores are plotted so that positive scores signify that more money was transferred to the HLA-similar trustee and a negative score denotes that more was transferred to the HLA-dissimilar trustee.

The multilevel linear regression model including the HLA-difference scores as covariate also revealed that the HLA similarity between the truster and the trustee was not related to how much money the trustee was entrusted with (see Table 1). The second model including the HLA-difference scores and pleasantness difference scores as covariates revealed that a man’s body odour pleasantness significantly predicted how much money he was entrusted with. HLA similarity between the truster and trustee again had no effect on the amount of money that was transferred. The third model revealed that the effect of odour pleasantness on trustworthiness decisions remained when controlling for odour intensity. Odour intensity and HLA-similarity had no effect on the amount of money that was transferred. The relationship between HLA-similarity, rated pleasantness and the amount transferred to the trustee in the Trust Game is depicted in Fig. 2.

**Table 1:** Results from multilevel linear regression analyses estimating differences in amount transferred in the trust game (N = 90).

Predictor	*Estimate*	*SE*	95%CI [LB, UB]		*t*	*df*	*p*-value
Model 1							
HLA-difference-score	−0.020	0.162	[−0.339,	0.300]	−0.122	163.91	0.903
Model 2							
Predictor	*Estimate*	*SE*	95% CI[LB, UB]	*t*	*df*	*p*-value	
HLA-difference-score	0.034	0.142	[−0.246,	0.315]	0.244	162.76	0.807
Pleasantness-difference-score	5.425	0.764	[3.917,	6.933]	7.105	162.27	<0.001***
Model 3							
Predictor	*Estimate*	*SE*	95% CI[LB, UB]	*t*	*df*	*p*-value	
HLA-difference-score	−0.003	0.142	[−0.284,	0.278]	−0.020	161.58	0.984
Pleasantness-difference-score	5.908	0.802	[4.325,	7.492]	7.369	158.83	<0.001***
Intensity-difference-score	1.369	0.741	[−0.096,	2.835]	1.849	127.06	0.067

Notes: Estimate, unstandardised regression coefficients; SE, standard error; CI, confidence interval; LB, lower bound; UB, upper bound.

Akaike’s information criterion (AIC) shows that Model 3 is the best fitting, parsimonious model (AIC: 840.093; compared to 844.732 in the second best Model 2 and 890.206 in Model 1).

### Allocation game

Paired t-tests revealed that there was no significant difference between the amount of money transferred to the HLA-similar and HLA-dissimilar donor (*M*_*similar*_ = 3.506, *SD*_*similar*_ = 1.266, *M*_*dissimilar*_ = 3.494, *SD*_*dissimilar*_ = 1.266, *t* = 0.042, *df* = 89, *p* = 0.967).

Likewise, the multilevel linear regression model including the HLA-difference scores as covariate revealed that the HLA similarity between the participant and the receiver did not significantly predict how the money was distributed (see Table 2). The second model including the HLA-difference scores and pleasantness difference scores as covariates revealed that a man’s body odour pleasantness significantly predicted how much money he was entrusted with. As before, HLA similarity between the participant and the receiver had no effect on the amount of money that was transferred in this donation game. The third model revealed that the effect of odour pleasantness on money distribution decisions remained when controlling for odour intensity, while odour intensity negatively predicted the amount of money that was transferred. HLA-similarity again had no effect on the amount of money transferred.

**Table 2:** Results from multilevel linear regression analyses estimating differences in amount transferred in the allocation game (*N* = 90).

Predictor	*Estimate*	*SE*	95% CI[LB, UB]		*t*	*df*	*p*-value
Model 1							
HLA-difference-score	−0.148	0.160	[−0.465,	0.169]	−0.923	155.65	0.357
Model 2							
Predictor	*Estimate*	*SE*	95% CI[LB, UB]	*t*	*df*	*p*-value	
HLA-difference-score	−0.088	0.136	[−0.357,	0.180]	−0.649	163	0.517
Pleasantness-difference-score	5.276	0.699	[3.895,	6.657]	7.544	163	<0.001***
Model 3							
Predictor	*Estimate*	*SE*	95% CI[LB, UB]	*t*	*df*	*p*-value	
HLA-difference-score	−0.086	0.136	[−0.355,	0.183]	−0.632	162	0.528
Pleasantness-difference-score	5.558	0.754	[4.069,	7.047]	7.372	162	<0.001***
Intensity-difference-score	0.849	0.848	[−0.826,	2.524]	1.001	162	0.318

Notes: Estimate, unstandardised regression coefficients; SE, standard error; CI, confidence interval; LB, lower bound; UB, upper bound.

Akaike’s information criterion (AIC) again shows that Model 3 is the best fitting model (AIC: 830.634; compared to 833.145 in the second best Model 2 and 881.131 in Model 1).

### Relationship between HLA-similarity and pleasantness

The model including the HLA-difference scores as covariate revealed that the odour pleasantness was not related to HLA-similarity (*Estimate* = −0.012; *standard error (SE)* = 0.014; 95%CI [−0.040, 0.017]; *t* = −0.809, *df* = 161.877; *p* = 0.420).

## Discussion

We tested the relative influence of kin selection and the beauty premium on trusting behaviour by using body odours as cues to trustworthiness decisions. We employed two economic games, a modified trust game and an allocation game in which participants were asked to divide 7 money units between two recipients who were represented solely by their body odour. In the trust game, the investors faced direct monetary consequences of their decisions, in the allocation game no consequences occurred for the investor. The body odours either stemmed from genetically similar (suggesting kin) or dissimilar (suggesting non-kin) men, as measured by similarity on the human leucocyte antigen complex (HLA). We found no evidence that HLA similarity influenced the participants decisions, neither in the trust game, nor in the allocation game. Instead, we found that the amount of money that was transferred was positively associated with the pleasantness of the recipient’s body odour.

From an evolutionary point of view, one might expect that people would invest more in their kin than in strangers. An intriguing way of assessing one’s relatedness to other group members is through body odours^3,17–19,34^. Specifically, body odours have been reported to portray information about an individual’s genotype at the HLA complex^25,35^. To directly test whether decisions about whom to trust and how to divide resources between two individuals are driven by odour-mediated cues to kinship, we presented body odours from individuals with a HLA complex that was highly similar or very dissimilar to the rater’s HLA complex.

Our results provide no evidence that odour-mediated HLA-similarity influences trustworthiness decisions, despite the fact that we maximized the likelihood of detecting an effect of HLA similarity. Specifically, we presented HLA-similar and dissimilar odours side by side and let participants distribute the money as they wished, while at the same time making it impossible to distribute the money equally. These results suggest that odour mediated cues to HLA similarity are too faint to influence trustworthiness decisions.

We found that raters transferred more money to the odour donors if they found their body odour pleasant. This parallels studies demonstrating a relationship between a man’s attractiveness and his apparent trustworthiness^29^. An explanation for why good-looking men are trusted more than men who are less good-looking is that attractiveness serves as an easily accessible cue to more complex and not easily accessible traits such as trustworthiness^36^. Others have emphasised the intrinsic value of attractiveness: There is an incentive to invest in good-looking men because of their high mate value^30^. Again others have noted that attractive individuals are also preferred as friends and collaborators, not just as mates^32,33^ suggesting that good-looking men are advantageous beyond mating motivations. In the present study we minimised the influence of mating motivation on trustworthiness decisions by including only men as odour donors and raters.

A pleasant body odour, just like a good looking face, may signal high partner value in general, because it might be a valid indicator of good health and low pathogen load^37^. A pleasant body odour may therefore predict longevity and, as a consequence, one might expect continued ability to extract resources from the environment which is valuable in multiple types of social partners. We note that having a pleasant body odour does not necessarily mean being a more cooperative or a more trustworthy partner in modern societies. It is likely, however, that pleasant-smelling body odours predicted a larger stream of material benefits in ancestral hunter-gatherer environments. Any strategies that help to choose the right partner most likely evolved in ancestral societies, and these same mechanisms may still produce preferences for pleasant-smelling and attractive cooperative partners in modern society^33^.

Interestingly, we found the same advantage for pleasant body odours in both the trust game and the allocation game, despite the fact that the games differed as to whether participants would face direct consequences from their decisions. We might have expected a larger influence of genetic similarity particularly in situations where trusting the right person has direct consequences for the investor, because fair reciprocation might more likely occur among kin. The fact that we found a comparable beauty premium in both games (and no influence of genetic resemblance in either of the games) further corroborates the idea that the beauty premium is a powerful heuristic which overrides other more subtle odour-mediated cues, such as HLA-resemblance.

We note that the evidence for HLA-mediated social behaviour is controversial. Also, although HLA similarity has been shown to be perceivable through olfaction, HLA similarity might be a somewhat limited and noisy measure for general genetic similarity. Notwithstanding these limitations, our results suggest that body odour pleasantness plays a more crucial role in social decisions such as whom to trust than odour mediated HLA-similarity.

In conclusion, we found that recipients with body odours that were rated as more pleasant by the investor received higher amounts of money than recipients whose body odours were rated less pleasant, irrespective of whether the participant faced direct consequences from his decision (trust game) or not (allocation game). We found no indication that HLA similarity influences such investment decisions. Together the present findings suggest that whether we find a body odour pleasant or not significantly influences our decisions on how to distribute resources and that this beauty premium possibly overwrites any potential influence of odour mediated cues to genetic resemblance.

## Methods

### Participants

Participants were recruited via advertisements on noticeboards, flyers and websites. Initially, 129 men ranging in age between 20 and 36 years agreed to participate in this study. Thirty-three acted as odour donors (mean age = 23.27, SD = 3.80) and 96 as odour raters (all male mean age = 23.41, SD = 3.71). All participants were Caucasian and of European descent (at least back to their grandparents). All experimental protocols were approved by the Ethics Committee of the Canton of Bern. All participants were treated in accordance with the ethical protocol approved by the Ethics Committee of the Canton of Bern and all provided written informed consent to take part in this study. Odour donors received a flat fee of 80 CHF, odour raters a flat fee of 60 CHF as compensation. These fees were supplemented by any earnings emerging from the trust game (between 14 and 28 CHF, see below). Each participant received his payment in cash in private at the end of the experiment.

### General procedure

The general procedure involved two phases. Phase I consisted of the blood sampling, HLA typing, HLA similarity calculation, odour collection and compliance interview. Phase II consisted of an experimental session in which raters were invited to play two rounds of a modified trust game (TG) and two rounds of an allocation game (AG), taking the role of the investor. Each rater was asked to divide a sum of money between two other players, represented only by their body odour. Subsequently, raters rated the male body odours for pleasantness and intensity on a visual analogue scale (1–100).

### Phase I

#### Blood sampling and HLA typing procedure

Eligible participants (129 men) were invited to the laboratory for venous blood sampling. Before blood sampling, participants read the study information and gave written informed consent. The participants’ blood samples (10 ml) were genotyped at the six loci that show the greatest variability^38^, three at Class I (HLA-A, HLA-B, HLA-C) and three at Class II (HLA-DRB1, HLA-DQA1, HLA-DQB1) using LinkSēq™ test kits (Linkage Biosystems^TM^). These test kits are based on real-time polymerase chain reaction (PCR) using allele-specific exponential amplification (sequence-specific primers). The resulting amplimers were subjected at end-point to a melting curve analysis to identify specific DNA based on melting temperature using SYBR® Green. Attribution of HLA-genotypes was done using SureTyper™ software. Ambiguities were resolved using alternative typing methods via routine HLA-typing.

#### Odour collection procedure

Odour donors (all male) were initially screened in a telephone interview for the required inclusion criteria: (a) aged between 17 and 40 years, (b) medication-free (for at least the 3 previous months), and (c) non-smoker.

The odour donors were requested to follow a strict schedule of dietary and behavioural restrictions while collecting their body odour (see supplementary material, Section A, for details). On the evenings of the sampling, before applying the cotton axillary pads to their left and right armpits, odour donors were instructed to take a shower with the non-perfumed soap supplied in the material package. Then donors fixed cotton pads (Ebelin cosmetic pads, DM-drogerie markt, www.dm-drogeriemarkt.de) to both armpits using 3 M Micropore surgical tape. Donors collected body odour on three consecutive nights, resulting in six odour pads per donor.

After odour collection, the pads were stored in separate sealable plastic bags and were frozen at −30 °C until use^39^.

#### Compliance interview and donor dropouts

When returning their body odour samples to the lab, donors were asked a series of questions in a structured face-to-face interview, assessing how long they had worn their axillary pads, whether they had complied with the dietary and behavioural restrictions (see supplementary material, Section B). We followed and slightly adapted the structured interview used by Gildersleeve and colleagues^40^.

Of the 33 men initially participating as odour donors, three withdrew without giving a reason, two took medication during the odour collection, one had problems with the blood sampling and three were excluded because they violated the behavioural restrictions. The final sample of odour donors hence consisted of 24 men.

#### HLA similarity

For each rater, we pre-selected 2 HLA-similar and 2 HLA-dissimilar body odour pads. To do so, we first calculated an HLA-Similarity-Index for each rater-donor pair by adding up the shared alleles on the six loci (HLA-A, HLA-B, HLA-C, HLA-DRB1, HLA-DQA1, HLA-DQB1). In a second step, for each male rater, we chose as HLA-dissimilar donors those with the lowest values of HLA-similarity and as HLA-similar donors those with the highest values of HLA-similarity. On average, raters shared 6.18 alleles with HLA-similar donors (SD = 0.116) and 0.88 alleles with the HLA-dissimilar donors over all the six HLA loci (SD = 0.061). These numbers compare nicely with the shared alleles in other studies^41–43^.

### Phase II

Phase two consisted of an experimental session in which raters were invited to play two rounds of a modified trust game (TG) and two rounds of an allocation game (AG). Following this, raters rated the body odours for pleasantness and intensity.

To prepare for the experimental session, odour raters were asked to refrain from eating and drinking caffeinated or alcoholic beverages for 1 h prior to testing, as these activities are known to affect smelling ability. Axillary pads from each donor were thawed three hours before the test and were placed in a 500 ml opaque glass jar. Three research assistants smelled the pads and confirmed that none was contaminated with extraneous odours (e.g., perfume, smoke). Only left-arm odour samples were used.

Raters were provided with clean white cotton gloves (carefully washed with non-perfumed washing detergent), which they were to wear during the whole session. To make their decisions, participants were asked to carefully open the lid of the glass jar containing the respective odour pad and to sniff it (taking a normal breath) without touching the rim of the glass. Sniffing time was not restricted.

#### Trust game

The Trust Game is illustrated in Fig. 1. For each odour rater, we pre-selected two HLA-similar and two HLA-dissimilar body odour pads (see Section *HLA Similarity*). Each rater played two rounds of a trust game, taking the role of the investor. For this he received 7 monetary units (MUs) per round which he was asked to divide between two trustees, represented only by their body odour. One trustee was HLA similar, the other was HLA dissimilar. For each round, the two odours were presented side by side in their respective jars and participants were asked to smell each body odour and decide how they were to divide the 7 MUs. Following distributions of the MUs were possible: 0:7, 1:6, 2:5, 3:4, 4:3, 5:2, 6:1, 7:0. Raters were informed that the invested sum would be quadrupled and that each trustee (odour donor) could either back-transfer half of the received amount to the truster (rater) or keep all to himself. The raters were also told that the trustees’ decisions (i.e., whether each trustee would reciprocate or keep all to himself) were pre-recorded and that both trustees made their decision independently of each other without knowing what the other trustee decided. Trustees were asked whether they would reciprocate or not when they returned the pads to the laboratory. The trustees’ decisions were unknown to the investors until the end of the experiment and investors could not revise their decisions.

#### Allocation game

Following the trust game, participants played two rounds of an allocation game. For this they received another 7 monetary units (MUs) per round which they were asked to divide between two recipients, again represented only by their body odour. One recipient was HLA similar, the other was HLA dissimilar. The sniffing procedure was the same as in the TG. Note that the pairings of the odours were different than in the TG but were made up from the same four odour pads as in the trust game (two HLA-similar and two HLA-dissimilar). Following distributions of the MUs were possible: 0:7, 1:6, 2:5, 3:4, 4:3, 5:2, 6:1, 7:0.

#### Body odour rating

After the TG and the AG, odour raters rated the body odour of the four pads used in the social decision tasks. The order of pads was randomized for each rater. Odour raters were asked to rate the body odour samples on a visual analogue scale (0–100) for pleasantness and intensity. If a rater found any of the samples too weak to assess, he was asked to select “I cannot smell the sample” instead of using the rating scales. Sniffing time was not restricted. After assessing the odour of a pad, raters were asked to sniff at a neutral pad to go back to a neutral reference.

### Ethics

The study was approved by the Ethics Committee of the Canton of Bern and was conducted according to the principles expressed in the Declaration of Helsinki.

## Supplementary information

Supplementary Info

Dataset 1.

## Data Availability

All data generated or analysed during this study are included as Supplementary Information file.
