# Supplementary material for: Pleasant body odours, but not genetic similarity, influence trustworthiness in a modified trust game
Source: Sci Rep. 2020 Feb 25;10:3388. doi: 10.1038/s41598-020-60407-6 (PMC7042344; doi:10.1038/s41598-020-60407-6)
Supplement: Supplementary file 1 — Supplementary Info [file 41598_2020_60407_MOESM1_ESM.pdf]

Supplementary Material:

Pleasant body odours, but not genetic similarity, influence trustworthiness in a modified trust game

Janek S. Lobmaier, Fabian Probst, Urs Fischbacher, Urs Wirthmüller, & Daria Knoch

A) Details on the odour collection procedure

Odour donors (all male) received a package of experimental material (white axillary cotton pads each packed in separate sealed plastic bags, a T-shirt, non-perfumed soap, shampoo, and laundry detergent).
Participants received the material in separate sealed bags. Following (Gildersleeve et al., 2012), the odour donors were asked to follow a schedule of dietary and behavioural restrictions on the day prior to the sampling and during the sampling nights. They were instructed to refrain from (a) using perfumes, deodorants, antiperspirants and perfumed shower gels; (b) eating meals containing garlic, onion, chilli, pepper, vinegar, blue cheese, cabbage, radish, fermented milk products, and marinated fish; (c) drinking alcoholic beverages or using drugs, and (d) smoking. Additionally, they were asked to refrain from strenuous physical activities, sexual intercourse, or sleeping in the same bed with a partner or pet during odour collection.
We used Ebelin cosmetic pads (elliptical in shape, approximately 9 x 7cm, DM-drogerie markt, www.dm-drogeriemarkt.de). These pads were the same as the ones that have been used in previous studies (e.g., Fialova et al., 2016).
On the evening of the sampling, before applying the cotton axillary pads to their left and right armpits, odour donors were instructed to take a shower with the non-perfumed soap supplied in the material package. The odour donor fixed the cotton pads to both armpits using 3M Micropore surgical tape. The pads were elliptical in shape, approximately 7-9 cm at their longest axis. Each pad was worn for one night. Men repeated the procedure for a total of three consecutive nights using new pads every night. To avoid odour contamination from donors' clothes or from the immediate environment, the donors were asked to wear the T-shirt supplied in the material pack (previously washed without washing powder) as the first layer of clothes and to previously wash all bedclothes with the supplied non-perfumed laundry detergent.
Participants returned the pads in separate sealed plastic bags after use. From the time-point the pads were returned to the lab onwards the lab-managers always wore clean white cotton gloves (carefully washed with non-perfumed washing detergent) and always used tweezers/forceps when handling the pads.

B) Structured compliance interview

We performed a structured face-to-face compliance interview with each donor when he returned his pads. We adopted the structured interview used by Gildersleeve et al. (2012) and included following questions:

- How long did you wear the cotton pad (duration in hours and minutes) each night (separate answers for Night 1, Night 2, Night 3)
- Did you experience any kind of complications? (open question)
- Did you use any of the following cosmetic products during the odour collection period? (Perfume, deodorant, antitranspirant, shower gel, body lotion, shampoo, facial tonic, conditioner, make-up, sports cream, facial cream) (separate answers for Night 1, Night 2, Night 3)
- In the evening, before applying the pads, did you wash your body and hair using the non-perfumed washing products supplied in the material pack?
- Did you wash your bedding (pillow case, sheets), your pyjama and your washing towels using the washing detergent supplied in the material pack?
- Did anybody lie or sleep in your bed (human being or animal) during the days/nights of odour collection (separate answers for Night 1, Night 2, Night 3)?
- Did you eat any of the following foods (including the day before odour collection): garlic, onions, vinegar, hot spices (e.g., chili/curry), strong cheese, cabbage, radish, fermented dairy products (whey, buttermilk, yoghurt, cottage cheese), marinated/smoked fish, seafood, asparagus, celeriac (separate answers for Day 1, Day 2, Day 3, Day 4)
- Did you ever smoke or sit in a smoking lounge during the period of odour collection?
- How many cigarettes did you smoke on Day 1 / Day 2 / Day 3 / Day 4?
- Did you take any medication (including vitamins) during the four days?
- Which medication (and how much) did you take on Day 1 / Day 2 / Day 3 / Day 4?
- Did you play any intense sports? (Separate answers for Day 1 / Day 2 / Day 3 / Day 4).
- Did you take any kind of drugs (dependence causing substances)? (Separate answers for Day 1 / Day 2 / Day 3 / Day 4).
- Did you drink any alcohol? How much? (Separate answers for Day 1 / Day 2 / Day 3 / Day 4).
- Were you involved in any sexual activity? (Separate answers for Day 1 / Day 2 / Day 3 / Day 4).
- Did you ever forget to freeze the cotton pads immediately after odour collection? (Separate answers for Night 1 / Night 2 / Night 3).

References:

Gildersleeve, K. A., Haselton, M. G., Larson, C. M., & Pillsworth, E. G. (2012). Body odor attractiveness as a cue of impending ovulation in women: Evidence from a study using hormone-confirmed ovulation. *Hormones and Behavior, 61*(2), 157-166.
